# Association of the inflammatory balance of diet and lifestyle with colorectal cancer among Korean adults: a case-control study

**DOI:** 10.4178/epih.e2022084

**Published:** 2022-09-30

**Authors:** Shinyoung Jun, Jeonghee Lee, Jae Hwan Oh, Hee Jin Chang, Dae Kyung Sohn, Aesun Shin, Jeongseon Kim

**Affiliations:** 1 Department of Cancer Biomedical Science, National Cancer Center Graduate School of Cancer Science and Policy, Goyang, Korea; 2 Center for Colorectal Cancer, National Cancer Center, Goyang, Korea; 3 Department of Preventive Medicine, Seoul National University College of Medicine, Seoul, Korea; 4 Cancer Research Institute, Seoul National University, Seoul, Korea

**Keywords:** Diet, Lifestyle, Inflammation, C-reactive protein, Colorectal cancer

## Abstract

**OBJECTIVES:**

Dietary and lifestyle exposures may affect the risk of colorectal cancer (CRC) by promoting chronic inflammation. Therefore, we assessed the separate and joint associations of dietary and lifestyle inflammation scores (DIS and LIS, respectively) with CRC.

**METHODS:**

Data from 919 pathologically confirmed CRC cases and 1,846 age- and sex-matched controls recruited at the National Cancer Center Korea were analyzed. We calculated the DIS and LIS, which characterize the collective contributions of 19 dietary and 4 lifestyle factors, respectively, to systemic inflammation by applying weights based on high-sensitivity C-reactive protein. A higher score represented a higher balance of pro- to anti-inflammatory exposures. Unconditional logistic regression models were used to estimate odds ratios (ORs) and 95% confidence intervals (CIs) for CRC risk compared across the DIS and LIS tertile categories, with the lowest tertile as the reference group.

**RESULTS:**

The highest DIS tertile had significantly increased odds of having CRC (OR, 2.65; 95% CI, 2.10 to 3.36), and the odds increased with increasing DIS. The highest LIS tertile group had 1.28-fold higher odds of having CRC (95% CI, 1.03 to 1.58). In the cross-classification analysis, the odds of having CRC increased as the DIS and LIS jointly increased until the DIS reached the highest tertile, where the risk was very high (3-fold or more) regardless of the LIS.

**CONCLUSIONS:**

In conclusion, a higher balance of pro-inflammatory relative to anti-inflammatory dietary and lifestyle factors, especially dietary factors, was associated with higher CRC risk among Korean adults.

## INTRODUCTION

In Korea, colorectal cancer (CRC) was the third most common cancer diagnosed in both males and females and was the third and second leading cause of cancer death in males and females, respectively, in 2019 [1]. The high burden of CRC in Korea has been largely attributed to changes in lifestyle and diet accompanying socioeconomic development [2]. Many of these lifestyle and dietary factors are known to promote chronic inflammation [3], which may mediate the development of CRC [4]. Consequently, many efforts have been made to identify modifiable risk factors for chronic inflammation and ultimately to develop interventions to reduce the risk of CRC [5].

To capture the collective effect of various risk factors on chronic inflammation, several indexes have been developed [6]. Among these indexes, the dietary inflammation score (DIS) and lifestyle inflammation score (LIS) [7] are relatively novel and comprehensive given that the DIS and LIS (1) can jointly assess inflammation-related dietary and non-dietary lifestyle factors, (2) include components selected *a priori* based on the previous literature and biological plausibility, and (3) weight each component based on its association with a biomarker of systemic inflammation (i.e., *a posteriori* method). In addition, the DIS uses food-level information and thus can account for a variety of bioactive substances in whole foods and avoid bias arising from incomplete or inconsistent food composition databases. Nonetheless, food-based indexes such as the DIS should be modified when applied to different populations to represent cultural ways of eating and to enable tailored dietary recommendations [6].

The DIS and LIS were associated with CRC in case-control and prospective cohort studies in the United States [8,9], supporting the use of these indexes. However, the associations between these scores and CRC in Korean adults may differ from those in United States adults because Korean adults maintain a considerable level of traditional dietary patterns [10] and have relatively low metabolic risk [11]. The objective of this study was to estimate the inflammatory balance of dietary and lifestyle factors using the DIS and LIS in a case-control study at the National Cancer Center Korea and to assess the association of the DIS and LIS with CRC in Korean adults.

## MATERIALS AND METHODS

### Study population

**Figure 1:**
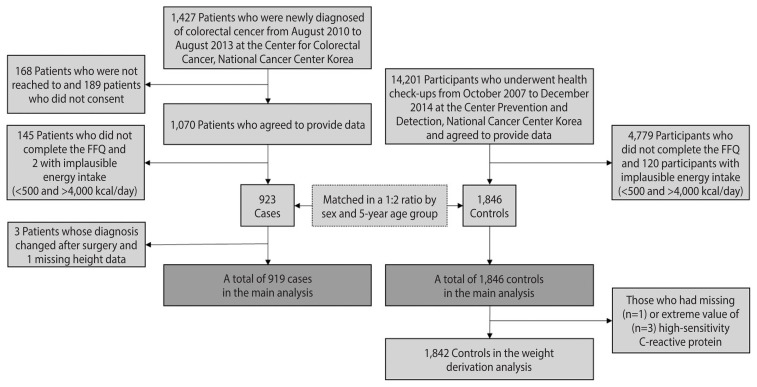
Selection of cases and controls. FFQ, food frequency questionnaire.

Case-control data were collected at the National Cancer Center Korea. Cases were recruited among patients who were newly diagnosed with adenocarcinoma of the colon or rectum based on endoscopic biopsies from August 2010 to August 2013 at the Center for Colorectal Cancer. Among the 1,427 patients contacted, 1,070 agreed to participate (75%). Controls were selected among those who underwent health check-ups at the Center for Cancer Prevention and Detection from October 2007 to December 2014 and were confirmed to be cancer-free. Participants with incomplete questionnaire or food frequency questionnaire (FFQ) data or implausible energy intake (<500 or >4,000 kcal/day) were excluded. Then, 1,846 controls were frequency-matched to 923 cases at a 2:1 ratio by sex and 5-year age group. A more detailed description of the sample can be found elsewhere [12]. Finally, we excluded 3 cases whose diagnoses changed after surgery and 1 case without height data, leaving 919 cases in the main analysis (Figure 1). The anatomical location of CRC was determined on the basis of the International Statistical Classification of Disease and Related Health Problems, 10th revision (ICD-10) and was categorized into the proximal colon (cecum, ascending colon, hepatic flexure, transverse colon, and splenic flexure), distal colon (descending colon, sigmoid-descending colon junction, and sigmoid colon), and rectum (rectosigmoid colon and rectum).

To obtain weights for the DIS and LIS components, data from 1,842 of the 1,846 controls who had information on high-sensitivity C-reactive protein (hs-CRP) were used (Figure 1); 1 participant was excluded due to the lack of hs-CRP information, and 3 due to hs-CRP levels above 9.2 mg/L, which were located to the far right of the other levels in the distribution and were suspected of having acute inflammation due to their levels being close to 10 mg/L [13].

### Data collection

Trained interviewers administered the general questionnaire and FFQ. Cases were contacted within a few days after the first hospital admission for surgery and were asked to report their lifestyle and habitual diet before their CRC diagnosis. The general questionnaire was structured and included questions about socio-demographic, lifestyle, and health characteristics. The semiquantitative FFQ used in this study was developed for Korean adults and validated against 12-day dietary records across seasons [14]. In brief, participants self-reported intake frequencies of 106 line items, typically comprising a group of similar individual foods, over a year. When all line items were disaggregated, a total of 410 individual foods were identified. Daily energy and nutrient intakes were estimated using Computer Aided Nutrition Analysis Program version 4 (Can-Pro 4.0; Korean Nutrition Society, Seoul, Korea).

Anthropometric measurements and a blood draw were conducted by trained health technicians on the same day (controls) and within a few days (cases) of the administration of the questionnaire. Body mass index (BMI) was calculated as weight in kilograms divided by the square of height in meters. When height or weight measurements were missing (n=34), self-reported height or body weight was used to calculate BMI. Plasma samples of both cases and controls were aliquoted and stored at −80°C for quantitative measurement until the hs-CRP level was measured using enzyme-linked immunosorbent assay kits according to the manufacturer’s instructions (E-EL-H5134; Elabscience; Houston, TX, USA). The coefficient of variation was <10%.

### Dietary inflammation score construction

We followed the procedure for calculating the DIS suggested by Byrd et al. [7]. First, we categorized the 410 food items into DIS food groups. The original DIS was developed for the United States population using the REasons for Geographic and Racial Differences in Stroke (REGARDS) dataset. Therefore, some food groups commonly consumed by Koreans were not included in any component. For example, Byrd et al. [7] excluded soybeans and shellfish due to infrequent consumption in the REGARDS participants (<1% consuming ≥1 serving/wk) and excluded whole grains due to inadequate measurement by the Block 98 FFQ [7]. However, Koreans frequently consume soybeans and shellfish. Many Koreans also regularly consume seaweed (e.g., gim) and fermented vegetables (e.g., kimchi) that have anti-inflammatory properties [15,16]. In addition, our FFQ queried the intake of whole grains, but was not suitable to distinguish between high-fat and low-fat dairy foods. Thus, to accurately reflect the diets of our subjects, we modified the original DIS food groupings, as described in Table 1. In summary, we included shellfish in the “fish” component and soybeans and soybean products in the “legumes” component, created the “whole grain” and “seaweed” components, and combined the “low-fat dairy” and “high-fat dairy” components. We also combined the “apples and berries” component and the “other fruits and real fruit juices” component given the small number of food items in each component and the similarity in their anti-inflammatory mechanisms, resulting in 18 food group components [7].

The next step was to calculate the supplement component score, the 19th DIS component. Our general questionnaire only had a section on medication use that included a subsection on the use of multivitamins, single vitamins, and calcium supplements (yes/no). Therefore, we categorized participants into supplement ‘users’ and ‘non-users’. As most supplemental nutrients and bioactive ingredients are considered to have anti-inflammatory properties, we assumed that supplement use would be an anti-inflammatory factor and assigned a score of “1” to ‘users’ and “0” to ‘non-users.’

### Lifestyle inflammation score construction

The LIS comprises 6 score components related to 4 lifestyle factors, including smoking, obesity, alcohol consumption, and physical activity. Byrd et al. [7] originally suggested 2 separate components for overweight (BMI ≥25 kg/m^2^) and obesity (BMI ≥30 kg/m^2^). However, few subjects had a BMI ≥30 kg/m^2^, and a BMI ≥25 kg/m^2^ is considered a cut-off for obesity in Asian countries [17]. Therefore, we combined the two components as “obesity” (BMI ≥25 kg/m^2^). Regarding alcohol consumption, we calculated the number of drinks consumed per week and categorized males consuming >14 drinks/wk and females consuming >7 drinks/wk as “heavy drinkers”, males and females not reporting any alcohol consumption as non-drinkers, and all others as “moderate drinkers” [7]. A drink was defined as 14 g of alcohol (e.g., 350 mL of draft beer, 300 mL of rice beer, 150 mL of wine, 90 mL of 20% soju, or 45 mL of 40% liquor) [18]. Physical activity levels were represented by metabolic equivalent of task (MET)-min/wk, measured using the short form of the International Physical Activity Questionnaire [19]. Subjects were categorized into tertiles based on the distribution of MET-min/wk among controls, and the highest and middle tertiles were labeled “heavily” and “moderately” physically active, respectively.

Each of the following components was binary (yes/no), and responses of ‘yes’ were coded as “1,” while ‘no’ was coded as “0.”: “current smoker”, “obese”, “heavy drinker”, “moderate drinker”, “heavily physically active”, and “moderately physically active”. The definitions of each category are presented in Table 1.

### Dietary inflammation score and lifestyle inflammation score calculation

Weights for the DIS and LIS components were determined using data from controls with reliable hs-CRP information so that the weights would represent the relationship of dietary and lifestyle factors with systemic inflammation prior to cancer development and be applicable to the general population. For the DIS, we first log-transformed the intake of each food group and standardized it by sex based on the distribution among controls. Then, we constructed a multivariate linear regression model including age, sex, case/control status, comorbidity (any history of self-reported cancers other than CRC, heart diseases, and diabetes mellitus), the regular use of aspirin or other non-steroidal anti-inflammatory drugs (NSAIDs; ‘once per week’ vs. ‘no use or less than once per week’), the use of hormone replacement therapy (among females), log-transformed total energy intake, and all 19 DIS and 6 LIS components. We kept most biological confounders suggested by Byrd et al. [7] unless they were not available in our data. The estimated β-coefficients represent the average change in the log-transformed hs-CRP concentration per 1 standard deviation increase in a food group-related DIS component, the consumption of any supplements, or the presence of an LIS component and were thus used as weights.

Finally, we multiplied each DIS and LIS component by its respective weight. The DIS was the sum of its 19 weighted component scores. The LIS was the sum of its 6 weighted component scores. A higher score of DIS and LIS represented a higher balance of pro- inflammatory to anti-inflammatory exposures.

### Statistical analysis

All analyses were conducted using SAS version 9.4 (SAS Institute Inc., Cary, NC, USA). Descriptive statistics for cases and controls are presented as the mean±standard deviation or number (%). The differences between the cases and controls were tested using the chi-square test for categorical variables and the Wilcoxon rank sum test for continuous variables with non-normal distributions.

Cases and controls were categorized into sex-specific DIS and LIS tertiles based on their distributions among controls. The associations of the DIS and LIS with overall CRC or site-specific CRC risk were assessed using unconditional logistic regression. Covariates in the logistic regression models were selected based on the previous literature [5,7] and biological plausibility with consideration of collinearity: age, sex, education level (‘high school graduate or less’ vs. ‘college graduate or more’), comorbidity, NSAID use, the use of hormone replacement therapy among females, first-degree relative history of CRC, total energy intake, and the DIS or LIS components. Trends across tertiles were tested by treating the order of the tertile as a continuous variable [20]. We also conducted sex-stratified analyses. Finally, to estimate the joint associations of the DIS and LIS with overall CRC risk, we conducted a cross-classification analysis by placing the participants in the joint first tertile of both scores as the reference group.

#### Sensitivity analyses

We conducted several sensitivity analyses to ensure that our weights for the DIS components were internally valid because the weighting method could overly influence the results [21]. First, we calculated the equal-weighted DIS by assigning positive or negative equal weights (i.e., ‘1’ or ‘−1’) to the DIS and LIS components according to the sign of weights derived from our data. Second, we constructed 19 DIS and 7 LIS components, as suggested by Byrd et al. [7]. Supplementary Material 1 shows the differences in the DIS food groupings between the study of Byrd et al. [7] and the current study. Last, we excluded participants with a first-degree relative history of CRC because they are more likely to be genetically predisposed to CRC [22] and may be less impacted by dietary and lifestyle factors or chronic inflammation.

### Ethics statement

The study protocol was approved by the Institutional Review Board (IRB) of the National Cancer Center Korea (IRB No. NCC 2022-0118). Informed consent was confirmed by the IRB.

## RESULTS

The demographics, lifestyle characteristics, medical history, and dietary intake of the cases and controls are presented in Table 2. Cases were less likely to have attained a tertiary education (i.e., college or more) and use aspirin or other NSAIDs regularly (i.e., once/wk) and were more likely to be heavy drinkers and have a relative history of CRC in first-degree relatives. The mean total energy intake was higher in cases than in controls, while no differences in the proportion of obese participants (i.e., BMI >25 kg/m^2^) were observed. The mean DIS and LIS were higher in cases than in controls.

The associations of both inflammation scores with CRC by anatomic site are presented in Table 3. When adjusted for age, education level, sex, comorbidity, the regular use of aspirin or other NSAIDs, the use of hormone replacement therapy among females, first-degree relative history of CRC, total energy intake, smoking, alcohol consumption, obesity, and physical activity (model 2), those in the highest DIS tertile had substantially higher odds of having CRC than those in the lowest DIS tertile (OR, 2.65; 95% CI, 2.10 to 3.36), and the odds increased with an increasing DIS (p<0.001). Among the 3 anatomic subsites of CRC (proximal colon, distal colon, and rectum), the associations with the DIS tended to be the strongest for rectal cancer. The LIS was less strongly associated with CRC than the DIS; those in the highest LIS tertile had 28% higher odds of having CRC (model 2; 95% CI, 1.03 to 1.59) than those in the lowest LIS tertile. When stratified by anatomic site, no significant associations were observed between the LIS and CRC subsites (model 2). Sex-stratified analyses suggested that the association of both scores with CRC may be stronger among females than among males (Supplementary Material 2).

In sensitivity analyses, the association of the equal-weighted DIS with CRC was slightly weaker than that of the weighted DIS (Supplementary Material 3). When we constructed the DIS, as was done for the United States population [7], the association of the DIS with CRC was even weaker than that of our equal-weighted DIS (Supplementary Material 4). The construction of the LIS, as was done for the United States population [7], by using the Asian-specific overweight and obesity cut-offs of BMI ≥23 to <25 and ≥25 kg/m^2^, respectively, resulted in non-significant associations with CRC (Supplementary Material 4). Moreover, excluding participants with a first-degree relative history of CRC did not alter the results (Supplementary Material 5).

Finally, we assessed the joint associations of the DIS and LIS with CRC (Table 4). In any LIS tertile, the odds of CRC tended to increase with an increasing DIS. In the first and second DIS tertiles, the odds of CRC had a tendency to increase with an increasing LIS; however, in the highest DIS tertile, no such trend was observed, with the odds of CRC being similar between the lowest (OR, 3.73; 95% CI, 2.43 to 5.71) and the highest (OR, 3.60; 95% CI, 2.38 to 5.43) LIS tertiles.

## DISCUSSION

To collectively account for the relatively small anti-inflammatory/pro-inflammatory effects of individual dietary and lifestyle factors, we calculated summary measures of the inflammatory balance of diet (DIS) and lifestyle (LIS). Both the DIS and LIS were positively associated with CRC, supporting the pathways linking environmental exposures, chronic inflammation, and CRC [4]. Our findings also suggest that pro-inflammatory diets may be a stronger risk factor for CRC than pro-inflammatory lifestyle factors among Korean adults.

In our study, the highest DIS tertile had 2.65-fold higher odds of having CRC than the lowest tertile, even after adjusting for potential confounders. The effect size was much larger than those reported in previous case-control and cohort studies utilizing the DIS in relation to CRC. When comparing the highest with the lowest quintile, a pooled analysis of 3 United States case-control studies reported an OR of 1.31 (95% CI, 0.98 to 1.75) for sporadic colorectal adenoma [8], a cohort study of middle-aged and older United States males and females reported a hazard ratio [HR] of 1.27 (95% CI, 1.19 to 1.35) for CRC [9], and another cohort study of postmenopausal United States females reported an HR of 1.07 (95% CI, 0.91 to 1.25) for CRC [23].

To rule out the possibility that the strong associations between the DIS and CRC in our study were an artifact of the scoring and weighting, we conducted several sensitivity analyses by constructing DIS components as similar as possible to those constructed in previous studies and by applying equal weighting. These sensitivity analyses yielded generally similar results, so we inferred that pro-inflammatory diets play a particularly powerful role in CRC development among Korean adults. Supporting our inference, in a previous analysis of data from our case-control study [24], the highest tertile of the dietary inflammatory index (DII), another index to quantify the inflammatory potential of diets, was associated with a fairly high CRC risk (OR, 2.16; 95% CI, 1.71 to 2.73) compared to the lowest tertile.

The association with CRC was stronger for the DIS than for the LIS in our study, which was unexpected given the higher weights assigned to the LIS components (i.e., stronger associations between the LIS components and log-transformed hs-CRP levels among controls). Even in the joint analysis, the DIS largely drove the associations. In previous United States studies, the LIS was more strongly associated with incident CRC than the DIS [23], although the associations of the DIS and LIS with all-cancer mortality were similar [25]. The relatively weak association between the LIS and CRC in our study may be partially attributable to the similar distributions of all LIS components, except for alcohol consumption, between the cases and controls. For example, obesity prevalence was indistinct between the cases and controls, although obesity is a major risk factor for CRC [26].

Another discrepancy between our findings and the findings from the United States studies [8,9,23] was the presence of stronger associations of both scores with CRC among males than females in the United States studies. According to previous findings, hypotheses focusing on biological or methodological differences by sex have been suggested [8]. The inconsistent findings in this Korean sample do not support innate biology-related hypotheses and rather suggest that different diets and lifestyles by sex and culture may determine the extent to which chronic inflammation-related risk factors impact CRC risk.

Of note, we adapted some DIS and LIS components to reflect the cultural context, which was a necessary and common step but may hinder the comparability of results. A few DIS components that were determined to be anti-inflammatory in the United States study [7] were classified as pro-inflammatory in our study (e.g., coffee and tea, dairy, poultry, legumes, and other vegetables), possibly due to the different food items included and the different food combinations consumed. For example, many Koreans often consume coffee with sugar and creamer [27], which may mitigate or even reverse the anti-inflammatory potential of coffee. A positive association between poultry and inflammatory biomarkers was also not surprising because it has been reported in the United Kingdom [28]. Future studies on the inflammatory balance of dietary factors should also consider the cultural context. In addition, 2 separate LIS components related to obesity status were combined given the differing cut-offs for obesity classification and the small proportion of individuals with a BMI greater than 30 kg/m^2^ in Korea [17]. Nonetheless, the results of the sensitivity analyses suggested that our scoring and weighting methods for the DIS and LIS successfully captured their collective contribution to CRC.

The strengths of our indexes include capturing small individual effects contributing to the same pathway and the intercorrelations among the components. In addition, the DIS and LIS components were weighted based on a systemic inflammation biomarker, hs-CRP, measured in our population, overcoming limitations related to the literature-based weights as in the case of the DII; in brief, the DII includes up to 45 individual food and nutrient components and uses weights derived from literature reviews [29]. The strengths of our data collection procedures include the use of histologically verified CRC information to reduce case misclassification and the implementation of a comprehensive questionnaire and clinical assessments that allowed us to control for potential confounding factors.

Major limitations arise from our case-control study design, including recall bias, selection bias, and a lack of temporality. To minimize recall bias, we asked the CRC patients to report their habitual diet and lifestyle prior to diagnosis, even though it is unlikely that newly diagnosed patients would have already changed their behaviors. Regardless of current disease status, the DIS and LIS components were based on self-report data and, thus, are subject to measurement error. Regarding selection bias, the controls were selected from people who visited the same hospital as the cases, so they were unlikely to have been from widely different regions. However, the controls who came for health check-ups may have been more health conscious than the cases, although the check-ups were supported by the Korea National Cancer Screening Program. To calculate the weights, we relied solely on hs-CRP, which is a strong predictor of CRC [30]; however, future studies may consider constructing a composite score for systemic inflammation using multiple biomarkers [7]. Last, we cannot rule out the possibility of residual confounding factors.

In conclusion, a higher balance of pro-inflammatory to anti-inflammatory dietary and lifestyle factors, particularly dietary factors, may be associated with a higher risk for CRC among Korean adults. In addition, our findings reiterate that major risk factors can vary across populations and support the use of indexes that are adaptable to different cultures. Diets that are known to exert generally small effects on carcinogenesis may play greater roles in a population with less variation in lifestyle. Although we could not determine causal relationships from this case-control study, our findings, as well as those from previous studies, suggest that reducing inflammation through dietary or lifestyle changes could potentially reduce the risk for CRC.

## Figures and Tables

**Table 1:** Components of the DIS and LIS and their descriptions and weights for this case-control study at the National Cancer Center Korea^1^

Components	Descriptions	Weights^2^
DIS components^3^		
Leafy greens and cruciferous vegetables	Spinach, cabbage or coleslaw, lettuce, watercress, kale, broccoli, bok choy, radish, other leafy greens, Kimchi or pickled radish	−0.19
Tomatoes	Tomatoes, tomato juice, tomato puree, and ketchup	−0.88
Deep yellow and orange vegetables and fruits	Peaches, persimmons, carrots, pumpkins	−0.11
Apples, berries, other fruits, and real fruit juices	Apples, pears, strawberries, watermelon, grapes, bananas, oranges, muskmelon, Korean melon, real fruit juice	−0.33
Other vegetables	Garlic, peppers, ginger, zucchini, celery, mushrooms, onion, green onion, soybean or mung bean sprouts, root vegetables, pickled vegetables	0.62
Legumes	Peas, soybeans, soy products	0.20
Fish and shellfish	White meat or dark meat fish, breaded fish cakes or fish sticks, shellfish	−0.36
Poultry	Chicken with and without skin	0.43
Red and organ meats	Beef, pork, organ meats	0.36
Processed meats	Ham, bacon, sausage	0.30
Added sugars	Sugar-sweetened soda, lemonade, jams, preserves, dried or canned fruit, syrup, honey, candy bars, chocolate, candy	0.24
Dairy	Milk, yogurt, cheese, ice cream	0.42
Coffee and tea	Coffee, green tea, herbal tea	0.16
Nuts and seeds	Peanuts, almonds, pine nuts, sesame seeds	−0.31
Other fats	Vegetable oil, mayonnaise, butter, margarine	0.04
Refined grains and starchy vegetables	White rice, noodles, ready-to-eat breakfast cereals, bread, cake, cookies, chips, crackers, biscuits, rice cakes, potato, sweet potato, starch	0.21
Whole grains	Brown rice, barley, sorghum, millet	−0.45
Seaweed	*Gim* (nori), *miyeok* (wakame), *dashima* (*konbu*)	−0.55
Supplement use	Consumers of multi- or single-vitamin and minerals vs. non-consumers	−0.67

LIS components^4^		
Heavy drinker	>14 drinks/wk for male and >7 drinks/wk for female	0.58
Moderate drinker	>0 to ≤14 drinks/wk for male and >0 to ≤7 drinks/wk for female	−0.65
Heavily physically active	The highest tertile of MET-min/wk (using cut-offs based on the distribution among controls)	−0.66
Moderately physically active	The middle tertile of MET-min/wk (using cut-offs based on the distribution among controls)	−0.29
Current smoker	Currently smoking tobacco	4.22
Obese BMI	BMI ≥25 kg/m^2^	4.08

DIS, dietary inflammation score; LIS, lifestyle inflammation score; MET, metabolic equivalent of task; BMI, body mass index.

1 Modified from Byrd et al. [8] to reflect the dietary intake and body weight status of Korean adults.

2 β-coefficient weights were obtained from a multivariable linear regression model according to the method used by Byrd et al. [8] in 1,842 controls with reliable data on high-sensitivity C-reactive protein (hs-CRP); The multivariable linear regression models represented the average change in a log-transformed hs-CRP concentration per 1 standard deviation increase in a DIS component or the presence of a LIS component; Covariates in the model included age, sex, case/control status, comorbidity (any history of cancer, heart diseases, or diabetes), regular use of aspirin or other non-steroidal anti-inflammatory drugs (≥ once/wk), hormone replacement therapy (among females), log-transformed total energy intake, and all the DIS and LIS components.

3 Each DIS component was log-transformed and standardized by sex based on the distribution among controls.

4 All LIS components were dummy-coded as “1” for the non-reference category and “0” for the reference category.

**Table 2:** Selected characteristics of participants in this case-control study at the National Cancer Center Korea

Characteristics	Cases (n=919)	Controls (n=1,846)^1^	p-value^2^
Demographics			
Age (yr)	56.6±9.7	56.1±9.1	0.243
Female	297 (32.3)	596 (32.3)	0.987
College graduate or more	232 (25.2)	941 (51.0)	<0.001

Medical history			
Aspirin or NSAID use (≥once/wk)	22 (2.4)	205 (11.1)	<0.001
Comorbidity^3^	123 (13.4)	201 (10.9)	0.055
Hormone therapy^4^	8 (2.7)	32 (5.4)	0.069
First-degree relative history of CRC	86 (9.4)	99 (5.4)	<0.001

Lifestyle			
Obese (BMI >25 kg/m^2^)	324 (35.3)	681 (36.9)	0.400
Current smoker	195 (21.2)	341 (18.5)	0.085
Alcohol consumption			<0.001
Non-drinker	343 (37.3)	650 (35.2)	
Moderate drinker	360 (39.2)	961 (52.1)	
Heavy drinker	216 (23.5)	235 (12.7)	
Physical activity (MET-hr/wk)^5^	44.6±49.2	56.1±83.3	0.271
LIS	1.9±2.7	1.7±2.7	<0.001

Dietary intake			
Total energy (kcal/day)	2,027.9±533.4	1,689.6±560.4	<0.001
DIS	0.6±1.7	−0.2±1.7	<0.001

Values are presented as mean±standard deviation or number (%); Percentages may not add up to 100% due to rounding.

NSAID, non-steroidal anti-inflammatory drug; BMI, body mass index; DIS, dietary inflammation score; LIS, lifestyle inflammation score; MET, metabolic equivalent of task.

1 Missing 43 controls for the education variable.

2 Differences between cases and controls were tested using the chi-square test for categorical variables and the Wilcoxon rank sum test for continuous variables with a non-normal distribution.

3 Any history of heart disease, diabetes mellitus, or cancer other than colorectal cancer.

4 Among females only (297 cases and 596 controls).

5 According to tertile cut-offs from the distribution of controls’ MET.

**Table 3:** Associations of the DIS and LIS with colorectal cancer in this case-control study at the National Cancer Center Korea, overall and by anatomic site^1^

Inflammation scores	Colorectal cancer		Proximal colon cancer		Distal colon cancer		Rectal cancer	
			
	Case/control, n	OR (95% CI)	Case/control, n	OR (95% CI)	Case/control, n	OR (95% CI)	Case/control, n	OR (95% CI)
Model 1								
DIS^2^								
T1	159/615	1.00 (reference)	34/615	1.00 (reference)	53/615	1.00 (reference)	71/615	1.00 (reference)
T2	273/616	1.52 (1.19, 1.94 )	44/616	1.23 (0.77, 1.99)	91/616	1.53 (1.06, 2.23)	131/616	1.66 (1.20, 2.31)
T3	487/615	2.72 (2.15, 3.43)	87/615	2.38 (1.54, 3.69)	150/615	2.56 (1.79, 3.66)	241/615	3.02 (2.22, 4.12)
p-for-trend		<0.001		<0.001		<0.001		<0.001
LIS^3^								
T1	273/636	1.00 (reference)	46/636	1.00 (reference	94/636	1.00 (reference)	129/636	1.00 (reference)
T2	280/647	1.02 (0.83, 1.25)	56/647	1.19 (0.79, 1.80)	76/647	0.78 (0.56, 1.09)	140/647	1.08 (0.83, 1.42)
T3	366/563	1.44 (1.17, 1.76)	63/563	1.51 (1.00, 2.26)	124/563	1.42 (1.05, 1.92)	174/563	1.46 (1.12, 1.90)
p-for-trend		<0.001		0.048		0.018		0.005

Model 2								
DIS^4^								
T1	159/615	1.00 (reference)	34/615	1.00 (reference)	53/615	1.00 (reference)	71/615	1.00 (reference)
T2	273/616	1.48 (1.16, 1.90)	44/616	1.19 (0.73, 1.92)	91/616	1.50 (1.03, 2.19)	131/616	1.63 (1.17, 2.27)
T3	487/615	2.65 (2.10, 3.36)	87/615	2.35 (1.51, 3.67)	150/615	2.53 (1.77, 3.63)	241/615	3.00 (2.19, 4.10)
p-for-trend		<0.001		<0.001		<0.001		<0.001
LIS^5^								
T1	273/636	1.00 (reference)	46/636	1.00 (reference)	94/636	1.00 (reference)	129/636	1.00 (reference)
T2	280/647	0.98 (0.79, 1.23)	56/647	1.12 (0.73, 1.70)	76/647	0.74 (0.53, 1.04)	140/647	1.00 (0.75, 1.33)
T3	366/563	1.28 (1.03, 1.59)	63/563	1.33 (0.88, 2.03)	124/563	1.22 (0.89, 1.67)	174/563	1.23 (0.93, 1.63)
p-for-trend		0.023		0.173		0.185		0.134

DIS, dietary inflammation score; LIS, lifestyle inflammation score; OR, odds ratio; CI, confidence interval; NSAID, non-steroidal anti-inflammatory drug.

1 The tertile cut-offs for DIS were ≤−0.91 (T1) and >0.56 (T3) among males and ≤−0.92 (T1) and >0.47 (T3) among females; The tertile cut-offs for LIS were ≤−0.29 (T1) and >3.43 (T3) among males and ≤−0.65 (T1) and >0 (T3) among females.

2 Covariates in the multivariable logistic regression model included age, sex, education (college graduate or more/high school graduate or less), comorbidity (any history of cancer, heart diseases, or diabetes), regular use of aspirin or other NSAIDs (≥ once/wk), hormone replacement therapy (among females), first-degree relative history of colorectal cancer (yes/no), and total energy intake.

3 Covariates in the multivariable logistic regression model included age, sex, education (college graduate or more/high school graduate or less), comorbidity (any history of cancer, heart diseases, or diabetes), regular use of aspirin or other NSAIDs (≥ once/wk), hormone replacement therapy (among females), and first-degree relative history of colorectal cancer (yes/no).

4 Covariates in the multivariable logistic regression model included age, sex, education (college graduate or more/high school graduate or less), comorbidity (any history of cancer, heart diseases, or diabetes), regular use of aspirin or other NSAIDs (≥ once/wk), hormone replacement therapy (among females), first-degree relative history of colorectal cancer (yes/no), total energy intake, smoking status (current/non-current), alcohol consumption (heavy/moderate/non-drinker), obesity (yes/no), and physical activity level (heavy/moderate/not active).

5 Covariates in the multivariable logistic regression model included age, sex, education (college graduate or more/high school graduate or less), comorbidity (any history of cancer, heart disease, or diabetes), regular use of aspirin or other NSAIDs (≥ once/wk), hormone replacement therapy (among females), first-degree relative history of colorectal cancer (yes/no), total energy intake, and equal-weighted DIS.

**Table 4:** Joint associations of the DIS and LIS with colorectal cancer in this case-control study at the National Cancer Center Korea^1^

Variables	LIS					

	T1		T2		T3	
		
	Case/control, n	OR (95% CI)	Case/control, n	OR (95% CI)	Case/control, n	OR (95% CI)
DIS						
T1	42/221	1.00 (reference)	57/239	1.15 (0.72, 1.84)	60/155	1.74 (1.08, 2.80)
T2	82/232	1.55 (0.99, 2.42)	87/207	1.86 (1.19, 2.90)	104/177	2.45 (1.57, 3.83)
T3	149/183	3.73 (2.43, 5.71)	136/201	2.97 (1.93, 4.55)	202/231	3.60 (2.38, 5.43)

DIS, dietary inflammation score; LIS, lifestyle inflammation score; OR, odds ratio; CI, confidence interval; NSAID, non-steroidal anti-inflammatory drug.

1 The tertile cut-offs for DIS were ≤−0.91 (T1) and >0.56 (T3) among males and ≤−0.92 (T1) and >0.47 (T3) among females; The tertile cut-offs for LIS were ≤−0.29 (T1) and >3.43 (T3) among males and ≤−0.65 (T1) and >0 (T3) among females; Covariates in the multivariable logistic regression model included age, sex, education (college graduate or more/high school graduate or less), comorbidity (any history of cancer, heart diseases, or diabetes), regular use of aspirin or other NSAIDs (≥ once/wk), hormone replacement therapy (among females), first-degree relative history of colorectal cancer (yes/no), and total energy intake; p-for-interaction was 0.026.

## SUPPLEMENTARY MATERIALS

Supplementary Material 1. Comparison of the DIS components between Western-focused and Korean-focused studies^1^

Supplementary Material 2. Sex-stratified associations of the DIS and LIS with colorectal cancer in a case-control study at the National Cancer Center Korea

Supplementary Material 3. Associations of the equal-weighted DIS with colorectal cancer in a case-control study at the National Cancer Center Korea^1^

Supplementary Material 4. Associations of the DIS and LIS calculated according to Byrd et al. [1] with colorectal cancer in a case-control study at the National Cancer Center Korea, overall and by anatomic site^1^

Supplementary Material 5. Associations of the DIS and LIS with colorectal cancer among those without family history of colorectal cancer in a case–control study at the National Cancer Center Korea^1^
