# Supplementary material for: Association of the inflammatory balance of diet and lifestyle with colorectal cancer among Korean adults: a case-control study
Source: Epidemiol Health. 2022 Sep 30;44:e2022084. doi: 10.4178/epih.e2022084 (PMC10089705; doi:10.4178/epih.e2022084)
Supplement: Supplementary Material 1. — Comparison of the DIS components between Western-focused and Korean-focused studies1 [file epih-44-e2022084-Supplementary-1.docx]

**Supplemental Material 1. Comparison of the DIS components between Western-focused and Korean-focused studies^1^**

|  | **Western-focused**  [1] | | **Korean-focused** | |
| --- | --- | --- | --- | --- |
|  | **Components (weights)** | **Descriptions** | **Components (weights)** | **What we added** |
| **Anti-inflammatory** | Leafy greens and cruciferous vegetables (-0.14) | Spinach, cabbage or coleslaw, lettuce, watercress, kale, broccoli | Leafy greens and cruciferous vegetables (-0.19) | Bok choy, radish, **Kimchi, pickled radish** |
|  | Tomatoes (-0.78) | Tomatoes, tomato juice, tomato puree, ketchup | Tomatoes (-0.88) |  |
|  | Deep yellow or orange vegetables and fruit (-0.57) | Peaches, carrots, pumpkins | Deep yellow or orange vegetables and fruit (-0.11) | Persimmons, **sweet potato** |
|  | Apples and berries (-0.65) | Apples, pears, strawberry, apple juice | Apples, berries, other fruits, and real fruit juices (-0.33) | Korean melon |
|  | Other fruits and real fruit juices (-0.16) | Watermelon, grapes, banana, oranges, muskmelon, grape or orange juice |  |  |
|  | Fish (-0.08) | White meat or dark meat fish, breaded fish cakes or fish sticks | Fish and shellfish (-0.36) | **Shellfish** |
|  | Nuts (-0.44) | Peanuts, almonds, pine nuts | Nuts and seeds (-0.31) | **Sesame seeds, Perilla seeds** |
|  | Supplement score (-0.80) | Ranked score of supplemental intakes of each nutrient | Supplement use (-0.67) | Consuming multi- or single-vitamin and minerals vs. nonconsumer |
|  | — |  | Whole grains (-0.45) | Brown rice, barley, sorghum, millet |
|  | — |  | Seaweed (-0.55) | Gim (nori), miyeok (wakame), dashima (konbu) |
| **Mixed signal** | Other vegetables (-0.16) | Garlic, green peppers, zucchini, celery, mushrooms, onion | Other vegetables (0.62) | Red peppers, green onion, soybean or mung bean sprouts, ginger, **other pickled vegetables** |
|  | Legumes (-0.04) | Peas | Legumes (0.20) | Soybeans, tofu, soy milk**,** starchy root vegetables, **soy paste (Doenjang)** |
|  | Poultry (-0.45) | Chicken with and without skin | Poultry (0.43) | **Chicken broth** |
|  | High-fat dairy (-0.14) | Whole milk, ice cream, cheese, yogurt | Dairy (0.42) |  |
|  | Low-fat dairy (-0.12) | Low-fat flavored milk, sorbet |  |  |
|  | Coffee and tea (-0.25) | Coffee, green tea, herbal tea | Coffee and tea (0.16) | Ssanghwa-cha, Ginseng tea |
| **Pro-inflammatory** | Red and organ meats (0.02) | Beef, pork, organ meats | Red and organ meats (0.36) |  |
|  | Processed meats (0.68) | Ham, bacon, sausage | Processed meats (0.30) |  |
|  | Added sugars (0.56) | Sugar-sweetened soda, lemonade, jams, preserves, dried or canned fruit, syrup, honey, candy bars, chocolate, candy | Added sugars (0.24) | Yuja tea, Sikhye |
|  | Refined grains and starch vegetables (0.72) | White rice, noodles, ready-to-eat breakfast cereals, bread, cake, cookies, chips, crackers, biscuits, potato, sweet potato, starch | Refined grains and starch vegetables (0.21) | Rice cakes |
|  | Other fats (0.31) | Vegetable oil, mayonnaise, butter, margarine | Other fats (0.04) | Suet |

^1^ Food items in bold were excluded in a sensitivity analysis applying weights from Byrd et al. [1] given their ambiguous impacts on chronic inflammation.
