# Supplementary material for: Association of the inflammatory balance of diet and lifestyle with colorectal cancer among Korean adults: a case-control study
Source: Epidemiol Health. 2022 Sep 30;44:e2022084. doi: 10.4178/epih.e2022084 (PMC10089705; doi:10.4178/epih.e2022084)
Supplement: Supplementary Material 2. — Sex-stratified associations of the DIS and LIS with colorectal cancer in a case-control study at the National Cancer Center Korea [file epih-44-e2022084-Supplementary-2.docx]

**Supplemental Material 2. Sex-stratified associations of the DIS and LIS with colorectal cancer in a case-control study at the National Cancer Center Korea**

| **Inflammation scores** | **Males** | | **Females** | |
| --- | --- | --- | --- | --- |
|  | **No. case/cont** | **OR (95% CI)** | **No. case/cont** | **OR (95% CI)** |
| **Model 1** |  |  |  |  |
| **DIS^2^** |  |  |  |  |
| **T1** | 116/417 | 1.00 (ref) | 43/198 | 1.00 (ref) |
| **T2** | 198/417 | 1.49 (1.11, 2.00) | 75/199 | 1.56 (0.998, 2.43) |
| **T3** | 308/416 | 2.14 (1.61, 2.85) | 179/199 | 4.17 (2.76, 6.31) |
| ***P*-for-trend** |  | <0.001 |  | <0.001 |
| **LIS^3^** |  |  |  |  |
| **T1** | 191/433 | 1.00 (ref) | 82/203 | 1.00 (ref) |
| **T2** | 189/426 | 1.04 (0.80, 1.34) | 91/221 | 1.04 (0.72, 1.51) |
| **T3** | 242/391 | 1.38 (1.07, 1.78) | 124/172 | 1.78 (1.23, 2.57) |
| ***P*-for-trend** |  | 0.013 |  | 0.002 |
| **Model 2** |  |  |  |  |
| **DIS^4^** |  |  |  |  |
| **T1** | 116/417 | 1.00 (ref) | 43/198 | 1.00 (ref) |
| **T2** | 198/417 | 1.43 (1.06, 1.93) | 75/199 | 1.53 (0.98, 2.39) |
| **T3** | 308/416 | 2.06 (1.54, 2.76) | 179/199 | 3.95 (2.61, 6.00) |
| ***P*-for-trend** |  | <0.001 |  | <0.001 |
| **LIS^5^** |  |  |  |  |
| **T1** | 191/433 | 1.00 (ref) | 82/203 | 1.00 (ref) |
| **T2** | 189/426 | 0.94 (0.71, 1.23) | 91/221 | 1.21 (0.82, 1.78) |
| **T3** | 242/391 | 1.22 (0.93, 1.60) | 124/172 | 1.63 (1.11, 2.40) |
| ***P*-for-trend** |  | 0.136 |  | 0.012 |

^1^ Case, cases; Cont, controls; CI, confidence interval; DIS, dietary inflammation score; LIS, lifestyle inflammation score; NSAID, nonsteroidal anti-inflammatory drug; OR, odds ratio. The tertile cutoffs for DIS were ≤-0.91 (T1) and >0.56 (T3) among males and ≤-0.92 (T1) and >0.47 (T3) among females. The tertile cutoffs for LIS were ≤-0.29 (T1) and >3.43 (T3) among males and ≤-0.65 (T1) and >0 (T3) among females.

^2^ Covariates in the multivariable logistic regression model included age, sex, education (college graduate or more/high school graduate or less), comorbidity (any history of cancer, heart disease, or diabetes), regular use of aspirin or other NSAIDs (≥ once/wk), hormone replacement therapy (among females), first-degree relative history of colorectal cancer (yes/no), and total energy intake.

^3^ Covariates in the multivariable logistic regression model included age, sex, education (college graduate or more/high school graduate or less), comorbidity (any history of cancer, heart disease, or diabetes), regular use of aspirin or other NSAIDs (≥ once/wk), hormone replacement therapy (among females), and first-degree relative history of colorectal cancer (yes/no).

^4^ Covariates in the multivariable logistic regression model included age, sex, education (college graduate or more/high school graduate or less), comorbidity (any history of cancer, heart disease, or diabetes), regular use of aspirin or other NSAIDs (≥ once/wk), hormone replacement therapy (among females), first-degree relative history of colorectal cancer (yes/no), total energy intake, smoking status (current/noncurrent), alcohol consumption (heavy/moderate/nondrinker), obesity (yes/no), and physical activity level (heavily/moderately/not active).

^5^ Covariates in the multivariable logistic regression model included age, sex, education (college graduate or more/high school graduate or less), comorbidity (any history of cancer, heart disease, or diabetes), regular use of aspirin or other NSAIDs (≥ once/wk), hormone replacement therapy (among females), first-degree relative history of colorectal cancer (yes/no), total energy intake, and equal-weighted DIS.
