# Supplementary material for: Association of the inflammatory balance of diet and lifestyle with colorectal cancer among Korean adults: a case-control study
Source: Epidemiol Health. 2022 Sep 30;44:e2022084. doi: 10.4178/epih.e2022084 (PMC10089705; doi:10.4178/epih.e2022084)
Supplement: Supplementary Material 3. — Associations of the equal-weighted DIS with colorectal cancer in a case-control study at the National Cancer Center Korea1 [file epih-44-e2022084-Supplementary-3.docx]

**Supplemental Material 3. Associations of the equal-weighted DIS with colorectal cancer in a case-control study at the National Cancer Center Korea^1^**

| **DIS** | **No. case/cont** | **OR (95% CI)** |
| --- | --- | --- |
| **Model 1^2^** |  |  |
| **T1** | 147/615 | 1 (ref) |
| **T2** | 289/613 | 1.77 (1.38, 2.26) |
| **T3** | 483/618 | 2.62 (2.06, 3.34) |
| ***P*-for-trend** |  | <0.001 |
| **Model 2^3^** |  |  |
| **T1** | 147/615 | 1 (ref) |
| **T2** | 289/613 | 1.75 (1.37, 2.25) |
| **T3** | 483/618 | 2.58 (2.02, 3.29) |
| ***P*-for-trend** |  | <0.001 |

^1^ Case, cases; Cont, controls; CI, confidence interval; DIS, dietary inflammation score; LIS, lifestyle inflammation score; NSAID, nonsteroidal anti-inflammatory drug; OR, odds ratio. Negative weights coded as ‘-1’ (i.e., considered anti-inflammatory) were assigned to 9 out of 19 DIS components (tomatoes; deep yellow and orange vegetables and fruits; apples, berries, other fruits, and real fruit juices; fish and shellfish; nuts; other fats; whole grains; seaweed; and supplement use) and 3 out of 6 LIS components (moderate drinker; heavily physically active; moderately and physically active. Positive weights coded as ‘1’ were assigned to the remaining components. The tertile cutoffs for equal-weighted DIS were ≤-2.20 (T1) and >1.76 (T3) among males and ≤-2.19 (T1) and >1.65 (T3) among females.

^2^ Covariates in the multivariable logistic regression model included age, sex, education (college graduate or more/high school graduate or less), comorbidity (any history of cancer, heart disease, or diabetes), regular use of aspirin or other NSAIDs (≥ once/wk), hormone replacement therapy (among females), first-degree relative history of colorectal cancer (yes/no), and total energy intake.

^3^ Covariates in the multivariable logistic regression model included age, sex, education (college graduate or more/high school graduate or less), comorbidity (any history of cancer, heart disease, or diabetes), regular use of aspirin or other NSAIDs (≥ once/wk), hormone replacement therapy (among females), first-degree relative history of colorectal cancer (yes/no), total energy intake, smoking status (current/noncurrent), alcohol consumption (heavy/moderate/nondrinker), obesity (yes/no), and physical activity level (heavily/moderately/not active).
