# Supplementary material for: Association of the inflammatory balance of diet and lifestyle with colorectal cancer among Korean adults: a case-control study
Source: Epidemiol Health. 2022 Sep 30;44:e2022084. doi: 10.4178/epih.e2022084 (PMC10089705; doi:10.4178/epih.e2022084)
Supplement: Supplementary Material 4. — Associations of the DIS and LIS calculated according to Byrd et al. [1] with colorectal cancer in a case-control study at the National Cancer Center Korea, overall and by anatomic site1 [file epih-44-e2022084-Supplementary-4.docx]

**Supplemental Material 4. Associations of the DIS and LIS calculated according to Byrd et al.** [1] **with colorectal cancer in a case-control study at the National Cancer Center Korea, overall and by anatomic site^1^**

| **Inflammation scores** | **Colorectal cancer** | | **Proximal colon cancer** | | **Distal colon cancer** | | **Rectal cancer** | |
| --- | --- | --- | --- | --- | --- | --- | --- | --- |
|  | **No. case /cont** | **OR (95% CI)** | **No. case /cont** | **OR (95% CI)** | **No. case /cont** | **OR (95% CI)** | **No. case /cont** | **OR (95% CI)** |
| **Model 1** |  |  |  |  |  |  |  |  |
| **DIS^2^** |  |  |  |  |  |  |  |  |
| T1 | 159/616 | 1.00 (ref) | 34/616 | 1.00 (ref) | 49/616 | 1.00 (ref) | 74/616 | 1.00 (ref) |
| T2 | 348/615 | 2.03 (1.60, 2.59) | 70/615 | 2.00 (1.28, 3.11) | 107/615 | 2.21 (1.52, 3.23) | 164/615 | 2.07 (1.50, 2.84) |
| T3 | 412/615 | 2.41 (1.90, 3.06) | 61/615 | 1.69 (1.07, 2.67) | 138/615 | 2.79 (1.93, 4.04) | 205/615 | 2.65 (1.94, 3.62) |
| *P*-for-trend |  | <0.001 |  | 0.041 |  | <0.001 |  | <0.001 |
| LIS^3^ |  |  |  |  |  |  |  |  |
| T1 | 295/639 | 1.00 (ref) | 54/639 | 1.00 (ref) | 96/639 | 1.00 (ref) | 139/639 | 1.00 (ref) |
| T2 | 299/627 | 1.01 (0.82, 1.24) | 51/627 | 0.99 (0.66, 1.49) | 91/627 | 0.95 (0.69, 1.30) | 149/627 | 1.04 (0.80, 1.36) |
| T3 | 325/580 | 1.19 (0.97, 1.46) | 60/580 | 1.23 (0.83, 1.83) | 107/580 | 1.20 (0.88, 1.63) | 155/580 | 1.22 (0.93, 1.59) |
| *P*-for-trend |  | 0.095 |  | 0.307 |  | 0.246 |  | 0.144 |
| Model 2 |  |  |  |  |  |  |  |  |
| DIS^4^ |  |  |  |  |  |  |  |  |
| T1 | 159/616 | 1.00 (ref) | 34/616 | 1.00 (ref) | 49/616 | 1.00 (ref) | 74/616 | 1.00 (ref) |
| T2 | 348/615 | 2.01 (1.58, 2.56) | 70/615 | 1.96 (1.25, 3.08) | 107/615 | 2.21 (1.51, 3.23) | 164/615 | 2.06 (1.49, 2.83) |
| T3 | 412/615 | 2.35 (1.85, 2.99) | 61/615 | 1.63 (1.03, 2.60) | 138/615 | 2.80 (1.93, 4.06) | 205/615 | 2.58 (1.88, 3.54) |
| *P*-for-trend |  | <0.001 |  | 0.064 |  | <0.001 |  | <0.001 |
| LIS^5^ |  |  |  |  |  |  |  |  |
| T1 | 295/639 | 1.00 (ref) | 54/639 | 1.00 (ref) | 96/639 | 1.00 (ref) | 139/639 | 1.00 (ref) |
| T2 | 299/627 | 0.95 (0.77, 1.18) | 51/627 | 0.91 (0.60, 1.38) | 91/627 | 0.87 (0.63, 1.21) | 149/627 | 0.98 (0.74, 1.29) |
| T3 | 325/580 | 1.06 (0.85, 1.32) | 60/580 | 1.10 (0.74, 1.65) | 107/580 | 1.07 (0.78, 1.47) | 155/580 | 1.07 (0.81, 1.41) |
| *P*-for-trend |  | 0.596 |  | 0.635 |  | 0.681 |  | 0.655 |

^1^ Case, cases; Cont, controls; CI, confidence interval; DIS, dietary inflammation score; LIS, lifestyle inflammation score; NSAID, nonsteroidal anti-inflammatory drug; OR, odds ratio. Please see Supplementary Table 2 for how we constructed DIS components for this analysis. LIS components were calculated exactly same as did Byrd et al. [1]. The tertile cutoffs for DIS were ≤-1.28 (T1) and >0.74 (T3) among males and ≤-1.35 (T1) and >0.60 (T3) among females. The tertile cutoffs for LIS were ≤0.05 (T1) and >0.89 (T3) among males and ≤-0.18 (T1) and >0.73 (T3) among females.

^2^ Covariates in the multivariable logistic regression model included age, sex, education (college graduate or more/high school graduate or less), comorbidity (any history of cancer, heart disease, or diabetes), regular use of aspirin or other NSAIDs (≥ once/wk), hormone replacement therapy (among females), first-degree relative history of colorectal cancer (yes/no), and total energy intake.

^3^ Covariates in the multivariable logistic regression model included age, sex, education (college graduate or more/high school graduate or less), comorbidity (any history of cancer, heart disease, or diabetes), regular use of aspirin or other NSAIDs (≥ once/wk), hormone replacement therapy (among females), and first-degree relative history of colorectal cancer (yes/no).

^4^ Covariates in the multivariable logistic regression model included age, sex, education (college graduate or more/high school graduate or less), comorbidity (any history of cancer, heart disease, or diabetes), regular use of aspirin or other NSAIDs (≥ once/wk), hormone replacement therapy (among females), first-degree relative history of colorectal cancer (yes/no), total energy intake, smoking status (current/noncurrent), alcohol consumption (heavy/moderate/nondrinker), obesity (yes/no), and physical activity level (heavily/moderately/not active).

^5^ Covariates in the multivariable logistic regression model included age, sex, education (college graduate or more/high school graduate or less), comorbidity (any history of cancer, heart disease, or diabetes), regular use of aspirin or other NSAIDs (≥ once/wk), hormone replacement therapy (among females), first-degree relative history of colorectal cancer (yes/no), total energy intake, and equal-weighted DIS.
