# Supplementary material for: Association of the inflammatory balance of diet and lifestyle with colorectal cancer among Korean adults: a case-control study
Source: Epidemiol Health. 2022 Sep 30;44:e2022084. doi: 10.4178/epih.e2022084 (PMC10089705; doi:10.4178/epih.e2022084)
Supplement: Supplementary Material 5. — Associations of the DIS and LIS with colorectal cancer among those without family history of colorectal cancer in a case–control study at the National Cancer Center Korea1 [file epih-44-e2022084-Supplementary-5.docx]

**Supplemental Material 5. Associations of the DIS and LIS with colorectal cancer among those without family history of colorectal cancer in a case–control study at the National Cancer Center Korea^1^**

| **Inflammation scores** | **No. case /cont** | **OR (95% CI)** |
| --- | --- | --- |
| **Model 1** |  |  |
| **DIS^2^** |  |  |
| **T1** | 146/581 | 1.00 (ref) |
| **T2** | 250/585 | 1.50 (1.16, 1.93) |
| **T3** | 437/581 | 2.71 (2.13, 3.45) |
| ***P*-for-trend** |  | <0.001 |
| **LIS^3^** |  |  |
| **T1** | 250/605 | 1.00 (ref) |
| **T2** | 252/608 | 1.00 (0.80, 1.24) |
| **T3** | 331/534 | 1.45 (1.17, 1.79) |
| ***P*-for-trend** |  | 0.001 |
| **Model 2** |  |  |
| **DIS^4^** |  |  |
| **T1** | 146/581 | 1.00 (ref) |
| **T2** | 250/585 | 1.46 (1.13, 1.88) |
| **T3** | 437/581 | 2.64 (2.06, 3.36) |
| ***P*-for-trend** |  | <0.001 |
| **LIS^5^** |  |  |
| **T1** | 250/605 | 1.00 (ref) |
| **T2** | 252/608 | 0.96 (0.76, 1.20) |
| **T3** | 331/534 | 1.27 (1.02, 1.59) |
| ***P*-for-trend** |  | 0.033 |

^1^ Case, cases; Cont, controls; CI, confidence interval; DIS, dietary inflammation score; LIS, lifestyle inflammation score; NSAID, nonsteroidal anti-inflammatory drug; OR, odds ratio. The tertile cutoffs for DIS were ≤-0.91 (T1) and >0.56 (T3) among males and ≤-0.91 (T1) and >0.52 (T3) among females. The tertile cutoffs for LIS were ≤-0.29 (T1) and >3.43 (T3) among males and ≤-0.65 (T1) and >0 (T3) among females.

^2^ Covariates in the multivariable logistic regression model included age, sex, education (college graduate or more/high school graduate or less), comorbidity (any history of cancer, heart disease, or diabetes), regular use of aspirin or other NSAIDs (≥ once/wk), hormone replacement therapy (among females), first-degree relative history of colorectal cancer (yes/no), and total energy intake.

^3^ Covariates in the multivariable logistic regression model included age, sex, education (college graduate or more/high school graduate or less), comorbidity (any history of cancer, heart disease, or diabetes), regular use of aspirin or other NSAIDs (≥ once/wk), hormone replacement therapy (among females), and first-degree relative history of colorectal cancer (yes/no).

^4^ Covariates in the multivariable logistic regression model included age, sex, education (college graduate or more/high school graduate or less), comorbidity (any history of cancer, heart disease, or diabetes), regular use of aspirin or other NSAIDs (≥ once/wk), hormone replacement therapy (among females), first-degree relative history of colorectal cancer (yes/no), total energy intake, smoking status (current/noncurrent), alcohol consumption (heavy/moderate/nondrinker), obesity (yes/no), and physical activity level (heavily/moderately/not active).

^5^ Covariates in the multivariable logistic regression model included age, sex, education (college graduate or more/high school graduate or less), comorbidity (any history of cancer, heart disease, or diabetes), regular use of aspirin or other NSAIDs (≥ once/wk), hormone replacement therapy (among females), first-degree relative history of colorectal cancer (yes/no), total energy intake, and equal-weighted DIS.

**Supplementary References**

1.Byrd DA, Judd SE, Flanders WD, Hartman TJ, Fedirko V, Bostick RM. Development and Validation of Novel Dietary and Lifestyle Inflammation Scores. J Nutr 2019;149:2206-2218.
